# Supplementary material for: Causal interplay between lactose intolerance and gut microbiota: a combined bidirectional Mendelian randomization and in vivo validation study
Source: Front Nutr. 2026 Jun 1;13:1803337. doi: 10.3389/fnut.2026.1803337 (PMC13265576; doi:10.3389/fnut.2026.1803337)
Supplement: Supplementary file 1 [file Data_Sheet_1.zip › supplementary materials/Forward/leave one out cross validation/ebi-a-GCST90027638.finngen_R12_E4_LACTONAS.pdf]

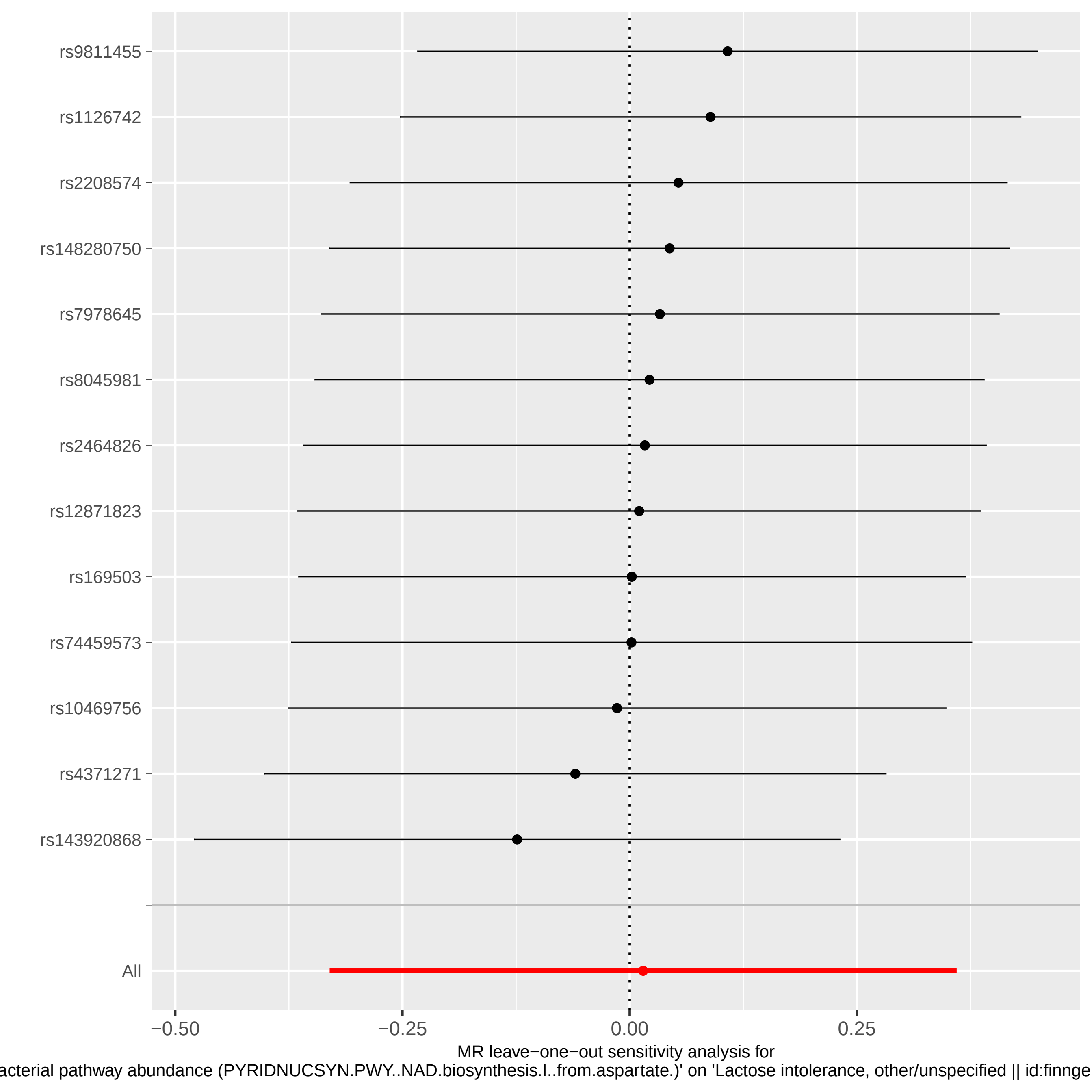
rs9811455
rs1126742
rs2208574
rs148280750
rs7978645
rs8045981
rs2464826
rs12871823
rs169503
rs74459573
rs10469756
rs4371271
rs143920868
All
−0.50
−0.25
0.00
0.25
MR leave−one−out sensitivity analysis for
acterial pathway abundance (PYRIDNUCSYN.PWY..NAD.biosynthesis.I..from.aspartate.)' on 'Lactose intolerance, other/unspecified || id:finnge
